# Cardiovascular adverse events associated with bispecific antibodies in relapsed or refractory B-cell non-Hodgkin lymphomas

**DOI:** 10.1186/s13045-026-01809-3

**Published:** 2026-05-26

**Authors:** Malak Munir, Ahmed Sayed, Dae Hyun Lee, Farrukh Awan, Sanam Ghazi, Jean Kim, Daniel Addison, Narendranath Epperla

**Affiliations:** 1 Department of Medicine, Cedars-Sinai Medical Center, Los Angeles, CA USA; 2 Department of Medicine, Rochester General Hospital, Rochester, NY USA; 3 Division of Cardiology, University of Pennsylvania, Thalheimer Center for Cardio- Oncology, Philadelphia, PA USA; 4 Division of Hematology and Oncology, University of Texas Southwestern Medical Center, Dallas, TX USA; 5 Cardio-Oncology Program, University of Texas Southwestern Medical Center, Dallas, TX USA; 6 Huntsman Cancer Institute, Division of Hematology and Hematologic Malignancies, University of Utah, Salt Lake City, UT USA

## Abstract

Bispecific antibodies (BsAb) approved for relapsed/refractory B-cell non-Hodgkin lymphomas (R/R NHL) redirect T-cells to malignant cells and have been associated with cardiovascular adverse events (CVAEs). We analyzed the FDA Adverse Event Reporting System (FAERS) data from December 2022 to September 2025 to characterize the safety of mosunetuzumab, glofitamab, and epcoritamab. Among 246,490 adverse event reports, 1,931 involved BsAbs. CVAEs occurred in 336 (17.4%) BsAb-associated reports of which 132 (39.3%) were fatal. The most frequent CVAEs were shock (3.2%), bleeding (3.0%), and hypotension (2.7%). As a class, BsAbs were associated with increased reporting of hypotension (aROR: 2.15, 95% CI = 1.48–3.13) and fatal CVAEs (aROR: 2.04, 95% CI = 1.60–2.60) compared to all other drugs in the FAERS database. Individual drugs showed distinct patterns: epcoritamab showed the strongest hypotension signal (aROR: 2.37, 95% CI = 1.55–3.62) and increased fatal CVAE reporting (aROR: 2.26, 95% CI = 1.72–2.98), while mosunetuzumab was associated with atrial fibrillation/flutter (aROR: 3.04, 95% CI = 1.53–6.03), supraventricular tachycardia (aROR: 2.72, 95% CI = 1.42–5.21), and tachyarrhythmias (aROR: 2.60, 95% CI = 1.44–4.72). CVAEs occurred earlier than non-CVAEs (median, 10 vs. 16 days, *P* < 0.001). Cytokine release syndrome occurred in 33.4% of BsAb-related adverse events, with hypotension most commonly overlapping (53.8%), while most arrhythmias occurred independently. These findings highlight cardiovascular toxicity signals associated with BsAbs and underscore the need for continued surveillance, risk stratification, and targeted cardiovascular assessment as these expand into broader clinical practice. However, consistent with the limitations of pharmacovigilance analyses, particularly the reliance on spontaneous adverse event reporting, these observations require confirmation in prospective studies.

**Supplementary Information:**

The online version contains supplementary material available at 10.1186/s13045-026-01809-3.

To the Editor,

Bispecific antibodies (BsAb) targeting CD20 on B-cells and CD3 on T-cells are approved for relapsed/refractory B-cell lymphomas in the second- and third-line settings [1]. Cardiovascular safety is an important consideration given their growing use in older patients with cardiovascular comorbidities and prior cardiotoxic exposures, including anthracyclines, BTK inhibitors, and CAR T-cell therapy [2, 3]. Individual BsAbs differ in dosing schedules and step-up strategies that may influence cardiovascular adverse event (CVAE) incidence and severity. However, systematic pharmacovigilance data comparing cardiovascular safety across these agents are lacking. We therefore conducted a comprehensive analysis of the FDA Adverse Event Reporting System (FAERS) to identify cardiovascular signals.

We analyzed FAERS reports from December 2022 through September 2025 listing mosunetuzumab, glofitamab, or epcoritamab as the primary suspect drug. CVAEs were identified using MedDRA queries (Table S1). Disproportionality analysis was performed using logistic regression to calculate adjusted reporting odds ratios (aRORs) for CVAEs, comparing BsAbs with all other FAERS reports. Expanded methods and sensitivity analyses are provided in the Supplementary Appendix.

We identified 1,931 BsAb reports among 246,490 total FAERS reports (0.8%). The cohort included 312 mosunetuzumab, 584 glofitamab, and 1,035 epcoritamab reports. The median age was 70 years (IQR: 59–77), and 59.1% (*n* = 1,142) were male. Diffuse large B-cell lymphoma (66.0%, *n* = 1274) was the most common underlying malignancy, followed by follicular lymphoma (11.3%, *n* = 218) (Table 1).

**Table 1:** Cohort characteristics

Characteristic	Overall	Mosunetuzumab	Glofitamab	Epcoritamab
Total reports, n (%)	1,931	312 (16.2%)	584 (30.2%)	1,035 (53.6%)
Age, median (IQR), years	70 (59-77)	68 (59.75-75)	64.5 (53.75-73)	72 (63-79)
Sex, n (%)				
Male	1142 (59.1)	171 (54.8)	359 (61.5)	612 (59.1)
Female	789 (40.9)	141 (45.2)	225 (38.5)	423 (40.9)
Reporter, n (%)				
Healthcare professional	1571 (81.4)	298 (95.5)	372 (63.7)	901 (87.1)
Consumer	227 (11.8)	7 (2.2)	179 (30.7)	41 (4.0)
Underlying malignancy, n (%)				
DLBCL	1274 (66.0)	30 (9.6)	435 (74.5)	809 (78.2)
Follicular lymphoma	218 (11.3)	114 (36.5)	17 (2.9)	87 (8.4)
Other B-cell lymphomas	34 (1.8)	1 (0.3)	29 (5)	4 (0.4)
B-cell lymphoma, subtype not specified	30 (1.6)	0 (0)	22 (3.8)	8 (0.8)
Lymphoma diagnosis not documented	375 (19.4)	167 (53.5)	81 (13.9)	127 (12.3)
Concomitant medications*, n (%)				
BTK inhibitor	30 (1.6)	8 (2.6)	12 (2.1)	10 (1.0)
Anthracycline	222 (11.5)	33 (10.6)	84 (14.4)	105 (10.1)
Cyclophosphamide	232 (12.0)	33 (10.6)	88 (15.1)	111 (10.7)
CAR T therapy	18 (0.9)	1 (0.3)	12 (2.1)	5 (0.5)

*Concomitant medications reflect all non-primary suspect drugs reported on the same FAERS report and may include prior, concurrent, or sequential therapies

CVAEs were reported in 336 (17.4%) BsAb reports, including 132 fatal cases (39.3%). The most frequently reported CVAEs were shock (*n* = 62, 3.2%), bleeding (*n* = 58, 3.0%), and hypotension (*n* = 52, 2.7%) (Table S2, Figure S1). Shock frequently co-occurred with infection (77.4%), whereas hypotension showed lower overlap with infection (32.7%) and greater overlap with cytokine release syndrome (CRS, 53.8%).

In the multivariable analysis, BsAbs were associated with increased reporting of hypotension (aROR = 2.15, 95% CI = 1.48–3.13, *n* = 52) and fatal CVAEs (aROR = 2.04, 95% CI = 1.60–2.60, *n* = 132) (Table S2). The highest fatality rates were observed for shock (69.4%, 43/62), heart failure (66.7%, 16/24), and bleeding (56.9%, 33/58) (Table S2, Figure S2). The occurrence of CVAEs was associated with increased reporting of fatal outcomes (aROR = 1.08, 95% CI = 1.003-1.16 for cardiac events; aROR = 1.10, 95% CI = 1.03–1.17 for vascular events), driven primarily by reports of heart failure (aROR = 1.40, 95% CI = 1.15–1.70), bleeding (aROR = 1.26, 95% CI = 1.11–1.42), and shock (aROR = 1.47, 95% CI = 1.30–1.66).

The proportion of CVAEs was similar across drugs: epcoritamab (18.7%, *n* = 194), glofitamab (16.1%, *n* = 94), and mosunetuzumab (15.4%, *n* = 48). Epcoritamab was associated with increased reporting of hypotension (aROR = 2.37, 95% CI = 1.55–3.62, *n* = 35) and fatal CVAEs (aROR = 2.26, 95% CI = 1.72–2.98, *n* = 86) (Fig. 1, Table S2). Mosunetuzumab was significantly associated with reports of atrial fibrillation/flutter (aROR = 3.04, 95% CI = 1.53–6.03, *n* = 9), supraventricular tachycardia (aROR = 2.72, 95% CI = 1.42–5.21, *n* = 10), and tachyarrhythmias (aROR = 2.60, 95% CI = 1.44–4.72, *n* = 12) (Fig. 1, Table S2). In contrast, glofitamab showed no statistically significant associations with CVAEs aside from fatal CVAEs (Fig. 1, Table S2). Notably, in disease-stratified analyses, fatal CVAEs were largely driven by reports in DLBCL (aROR = 3.02, interaction *P* = 0.016) (Table S3).

**Fig. 1:**
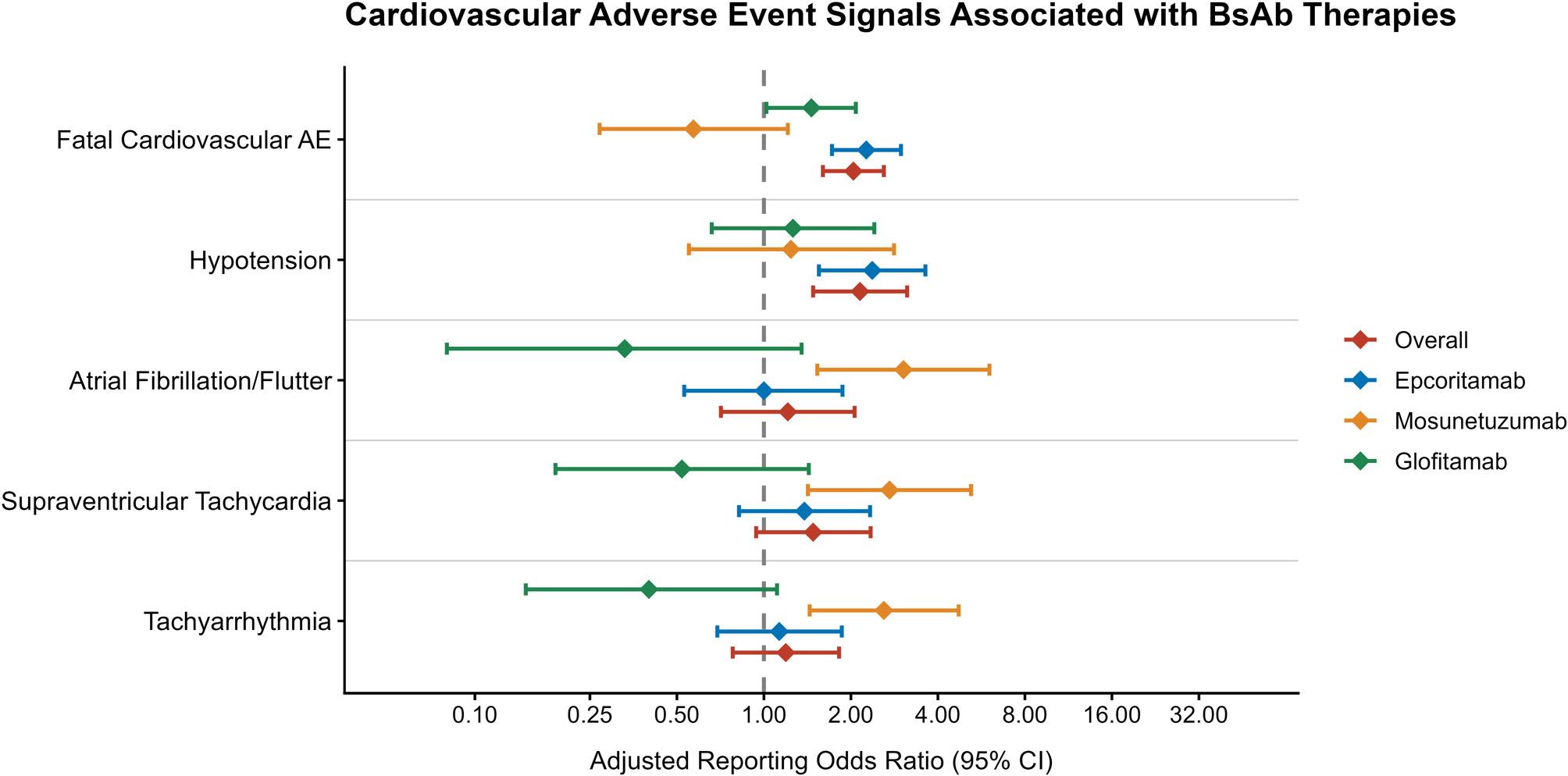
Cardiovascular adverse events reported with bispecific antibody therapies

CRS occurred in 644 (33.4%) of BsAb-related AEs. CRS was most frequently reported with epcoritamab (40.5%, *n* = 419) followed by glofitamab (26.5%, *n* = 155), and mosunetuzumab (22.4%, *n* = 70). Hypotension had the highest overlap with CRS (53.8%, 28/52), while heart failure overlapped in 45.8% of cases (11/24 cases) (Figure S3). Among mosunetuzumab-associated arrhythmias, atrial fibrillation/flutter co-occurred with CRS in 22.2% of cases (2/9), supraventricular tachycardia in 20.0% (2/10), and tachyarrhythmias in 16.7% (2/12).

Among reports with available timing data (48.2% of CVAEs, 45.8% of non-CVAEs), CVAEs occurred earlier than non-CVAEs (median = 10 days [IQR: 1–32], *n* = 162 vs. median = 16 days [IQR: 3–69], *n* = 731, *P* < 0.001). Among specific CVAEs with time-to-event data, hypotension occurred earliest (median = 4 days, IQR: 1–15, *n* = 28), followed by shock (median = 5 days, IQR: 1–19, *n* = 31), heart failure (median = 11 days, IQR: 5–30, *n* = 12), and bleeding (median = 13 days, IQR: 4–26, *n* = 29). Results were consistent in sensitivity analyses (Tables S3 -S5).

Our analysis identified several key findings. First, CVAEs were reported in approximately one in six BsAb-related AEs with disproportionately higher reporting of hypotension and fatal CVAEs. Second, individual BsAbs demonstrated distinct reporting patterns: epcoritamab showed the strongest signal for hypotension and fatal CVAEs, whereas mosunetuzumab was uniquely associated with arrhythmias, including atrial fibrillation, supraventricular tachycardia, and other tachyarrhythmias. Third, CVAEs tended to occur early and were frequently independent of CRS. Given the limited cardiovascular data from clinical trials [4], these real-world pharmacovigilance findings carry important clinical implications for monitoring and risk mitigation.

We observed differences in the reported cardiovascular signals between BsAbs and other T-cell therapies. In a prior FAERS analysis of CAR-T recipients, CVAEs were reported in approximately one in five cases, two-thirds of which overlapped with CRS, with a fatality rate of roughly one-third [5]. In contrast, we observed less overlap with CRS but a higher fatality rate. Previously reported signals of DIC and myocarditis with CAR-T and earlier BsAbs [5, 6] were not observed in our analysis. Hypotension, fatal CVAEs, and arrhythmias were the primary CVAE signals in our study, with distinct drug-specific patterns for epcoritamab and mosunetuzumab, findings supported by a meta-analysis of 37 clinical trials [7].

As BsAbs transition to broader use and are incorporated into first-line regimens alongside cardiotoxic therapies, [2, 8, 9] the potential for increased cardiovascular risk warrants careful surveillance, risk stratification, and proactive management. Prospective studies are needed to validate these pharmacovigilance signals and to establish monitoring and management strategies.

## Electronic Supplementary Material

Below is the link to the electronic supplementary material.

Additional file 1.

## Data Availability

The data used in this analysis are publicly available from the FDA Adverse Event Reporting System (FAERS) at: https://www.fda.gov/drugs/fdas-adverse-event-reporting-system-faers/fda-adverse-event-reporting-system-faers-latest-quarterly-data-files. Analysis code is available from the first author upon request.
