# Supplementary material for: Cardiovascular adverse events associated with bispecific antibodies in relapsed or refractory B-cell non-Hodgkin lymphomas
Source: J Hematol Oncol. 2026 May 26;19:33. doi: 10.1186/s13045-026-01809-3 (PMC13202884; doi:10.1186/s13045-026-01809-3)
Supplement: Supplementary file 1 — Additional file 1. [file 13045_2026_1809_MOESM1_ESM.docx]

# Supplementary Appendix

Contents

[Supplementary Appendix 1](#_Toc228478117)

[Supplemental Methods 2](#_Toc228478118)

[Methods 2](#_Toc228478119)

[Supplemental Tables and Figures 4](#_Toc228478120)

[Table S1. MedDRA Terms Used to Define Adverse Events 4](#_Toc228478121)

[Table S2. Proportions and Adjusted Reporting Odds Ratios of Cardiovascular Adverse Events Associated with BsAb Therapies. 18](#_Toc228478122)

[Table S3. Association Between BsAb Exposure and CVAEs Stratified by Disease. 20](#_Toc228478123)

[Table S4. Sensitivity Analysis Excluding Reports with Concomitant Cardiotoxic Drugs Reported. 21](#_Toc228478124)

[Table S5. Sensitivity Analysis Excluding Cases with Concurrent CRS 22](#_Toc228478125)

[Figure S1. Frequency of Cardiovascular Adverse Events Reported with Bispecific Antibodies (2022-2025). 23](#_Toc228478126)

[Figure S2. Fatality Rate of Cardiovascular Adverse Events Reported with Bispecific Antibodies. 24](#_Toc228478127)

[Figure S3. Overlap Between Cardiovascular Adverse Events and Cytokine Release Syndrome Reported with Bispecific Antibodies. 25](#_Toc228478128)

# Supplemental Methods

## Methods

***Data Source***In this retrospective pharmacovigilance study, we utilized the FDA Adverse Event Reporting System (FAERS), a global repository of post-marketing safety reports. We screened FAERS for reports from December 2022 to September 2025 containing BsAb therapies including mosunetuzumab, glofitamab, and epcoritamab. Reports were included if these agents were listed as the primary suspect drug. Duplicate reports for identical events were identified and only the most recent case version was retained.

Cardiovascular adverse events were identified using MedDRA Standardized Queries and System Organ Class searches for the following categories: heart failure and myocarditis; coronary disease and myocardial infarction; arrhythmias (tachyarrhythmia, supraventricular tachycardia, atrial fibrillation/flutter, ventricular tachyarrhythmia, ventricular tachycardia, ventricular fibrillation, bradyarrhythmia, QT prolongation, ventricular extrasystoles, sudden death); thromboembolic events (arterial and venous thromboembolism, cerebrovascular disease); vascular events (bleeding, shock, hypotension, hypertension, vasculitis); structural/inflammatory conditions (pericarditis, pericardial effusion, endocarditis, valvular disease); and other cardiovascular events (dyslipidemia, disseminated intravascular coagulation) (**Table S1**). Overlap between CRS and CVAEs was defined as co-occurrence of both events within the same adverse event report. A composite CVAE outcome was defined as the presence of any cardiovascular or vascular adverse event. Fatal CVAE was defined as any CVAE where the reported outcome was death.

***Statistical Analysis***

We performed a disproportionality analysis to detect signals of over-reporting for CVAEs associated with BsAb therapies compared to all drug-related reports included in the FAERS database. Adjusted reporting odds ratios (aRORs) and 95% confidence intervals were calculated using logistic regression models, adjusted for age, sex, disease category, concomitant BTK inhibitor, cyclophosphamide, CAR-T, and anthracycline exposure. Age was modeled using restricted cubic splines with 3 knots to allow for non-linearity.

To assess the association between adverse events and fatal outcomes among BsAb recipients, we fit logistic regression models with death as the outcome and individual adverse events as predictors, adjusting for age, sex, disease category, concomitant BTK inhibitor, cyclophosphamide, CAR-T, and anthracycline exposure. Odds ratios with 95% confidence intervals were estimated using average marginal effects, interpreted as measures of disproportionate reporting of fatal outcomes. Fatal outcomes were identified from the FAERS outcome field, indicating that death was the reported outcome of the adverse event.

Interaction terms between drug exposure and age, sex, and cardiovascular comorbidities were evaluated using likelihood ratio tests. Baseline cardiovascular comorbidity was identified from the FAERS indication field, defined as the presence of any cardiac or vascular disorder listed as an indication for concomitant (non-BsAb) medications in the same report. For significant interactions (P < 0.05), we calculated stratified odds ratios with 95% confidence intervals using average marginal effects to quantify how the association between BsAb exposure and adverse events differed across patient subgroups. The timing of onset of CVAE versus non-CVAE adverse events was compared using Wilcoxon rank-sum tests. All analyses were performed in R version 4.4.3.

**Limitations**

Our study has several limitations inherent to pharmacovigilance analyses. FAERS is a spontaneous reporting system subject to underreporting, reporting bias, and incomplete clinical information; causality cannot be definitively established. The database does not capture denominator data, preventing calculation of true incidence rates. Concomitant medication coding reflects drugs reported in association with the index event, not cumulative lifetime exposure. FAERS does not distinguish clinical trial reports from spontaneous post-marketing reports; drugs with larger or more recent development programs may have greater representation. The short post-marketing period means safety signals may evolve as more data accumulate. Finally, we could not account for differences in dosing schedules, CRS management protocols, or supportive care practices across institutions.

# Supplemental Tables and Figures

## Table S1. MedDRA Terms Used to Define Adverse Events

| Adverse Event | Preferred Terms Included |
| --- | --- |
| Heart failure | Acute left ventricular failure; Acute pulmonary oedema; Acute right ventricular failure; Cardiac asthma; Cardiac failure; Cardiac failure acute; Cardiac failure chronic; Cardiac failure congestive; Cardiac failure high output; Cardiogenic shock; Cardiohepatic syndrome; Cardiopulmonary failure; Cardiorenal syndrome; Chronic left ventricular failure; Chronic right ventricular failure; Congestive hepatopathy; Cor pulmonale; Cor pulmonale acute; Cor pulmonale chronic; Ejection fraction decreased; Hepatojugular reflux; Left ventricular failure; Low cardiac output syndrome; Neonatal cardiac failure; Obstructive shock; Pulmonary oedema; Pulmonary oedema neonatal; Radiation associated cardiac failure; Right ventricular ejection fraction decreased; Right ventricular failure; Ventricular failure |
| Myocarditis | Autoimmune myocarditis; Chronic myocarditis; Coxsackie myocarditis; Cytomegalovirus myocarditis; Enterovirus myocarditis; Eosinophilic myocarditis; Giant cell myocarditis; Hypersensitivity myocarditis; Immune-mediated myocarditis; Lupus myocarditis; Malarial myocarditis; Myocarditis; Myocarditis bacterial; Myocarditis helminthic; Myocarditis infectious; Myocarditis meningococcal; Myocarditis mycotic; Myocarditis post infection; Myocarditis septic; Myocarditis syphilitic; Myocarditis toxoplasmal; Radiation myocarditis; Viral myocarditis |
| Coronary disease | Acute cardiac event; Acute coronary syndrome; Acute myocardial infarction; Angina pectoris; Angina unstable; Anginal equivalent; Arteriosclerosis coronary artery; Arteriospasm coronary; Blood creatine phosphokinase MB abnormal; Blood creatine phosphokinase MB increased; Cardiac perfusion defect; Chronic coronary syndrome; Coronary angioplasty; Coronary arterial stent insertion; Coronary artery bypass; Coronary artery compression; Coronary artery disease; Coronary artery dissection; Coronary artery embolism; Coronary artery insufficiency; Coronary artery occlusion; Coronary artery reocclusion; Coronary artery restenosis; Coronary artery stenosis; Coronary artery surgery; Coronary artery thrombosis; Coronary brachytherapy; Coronary bypass stenosis; Coronary bypass thrombosis; Coronary endarterectomy; Coronary no-reflow phenomenon; Coronary ostial stenosis; Coronary revascularisation; Coronary steal syndrome; Coronary vascular graft occlusion; Coronary vascular graft stenosis; Diabetic coronary microangiopathy; ECG signs of myocardial ischaemia; External counterpulsation; Haemorrhage coronary artery; Heart-type fatty acid-binding protein increased; Ischaemic cardiomyopathy; Ischaemic mitral regurgitation; Kounis syndrome; Microvascular coronary artery disease; Myocardial hypoperfusion; Myocardial hypoxia; Myocardial infarction; Myocardial ischaemia; Myocardial necrosis; Myocardial reperfusion injury; Myocardial stunning; Papillary muscle infarction; Percutaneous coronary intervention; Periprocedural myocardial infarction; Post procedural myocardial infarction; Postinfarction angina; Prinzmetal angina; Silent myocardial infarction; Stress cardiomyopathy; Subclavian coronary steal syndrome; Subendocardial ischaemia; Troponin I increased; Troponin increased; Troponin T increased; Wellens' syndrome |
| Myocardial infarction | Acute cardiac event; Acute coronary syndrome; Acute myocardial infarction; Angina unstable; Blood creatine phosphokinase MB abnormal; Blood creatine phosphokinase MB increased; Coronary artery embolism; Coronary artery occlusion; Coronary artery reocclusion; Coronary artery thrombosis; Coronary bypass thrombosis; Coronary vascular graft occlusion; Heart-type fatty acid-binding protein increased; Kounis syndrome; Myocardial infarction; Myocardial necrosis; Myocardial reperfusion injury; Myocardial stunning; Papillary muscle infarction; Periprocedural myocardial infarction; Post procedural myocardial infarction; Postinfarction angina; Silent myocardial infarction; Troponin I increased; Troponin increased; Troponin T increased |
| Tachyarrhythmia | Accelerated idioventricular rhythm; Anomalous atrioventricular excitation; Arrhythmia supraventricular; Arrhythmic storm; Atrial fibrillation; Atrial flutter; Atrial parasystole; Atrial tachycardia; Cardiac fibrillation; Cardiac flutter; Congenital supraventricular tachycardia; Early repolarisation syndrome; Extrasystoles; Familial atrial fibrillation; Frederick's syndrome; Junctional ectopic tachycardia; Parasystole; Rhythm idioventricular; Sinus tachycardia; Supraventricular extrasystoles; Supraventricular tachyarrhythmia; Supraventricular tachycardia; Tachyarrhythmia; Torsade de pointes; Ventricular arrhythmia; Ventricular extrasystoles; Ventricular fibrillation; Ventricular flutter; Ventricular parasystole; Ventricular pre-excitation; Ventricular tachyarrhythmia; Ventricular tachycardia |
| Supraventricular tachycardia | Arrhythmia supraventricular; Atrial fibrillation; Atrial flutter; Atrial parasystole; Atrial tachycardia; Congenital supraventricular tachycardia; Familial atrial fibrillation; Frederick's syndrome; Junctional ectopic tachycardia; Sinus tachycardia; Supraventricular extrasystoles; Supraventricular tachyarrhythmia; Supraventricular tachycardia |
| Atrial fibrillation/flutter | Atrial fibrillation; Atrial flutter |
| Ventricular tachyarrhythmia | Accelerated idioventricular rhythm; Arrhythmic storm; Cardiac fibrillation; Early repolarisation syndrome; Parasystole; Rhythm idioventricular; Torsade de pointes; Ventricular arrhythmia; Ventricular extrasystoles; Ventricular fibrillation; Ventricular flutter; Ventricular parasystole; Ventricular pre-excitation; Ventricular tachyarrhythmia; Ventricular tachycardia |
| Ventricular tachycardia | Torsade de pointes; Ventricular tachycardia |
| Ventricular fibrillation | Ventricular fibrillation |
| QT prolongation | Electrocardiogram QT interval abnormal; Electrocardiogram QT prolonged; Long QT syndrome |
| Bradyarrhythmia | Accessory cardiac pathway; Adams-Stokes syndrome; Agonal rhythm; Atrial conduction time prolongation; Atrial escape rhythm; Atrial standstill; Atrioventricular block; Atrioventricular block complete; Atrioventricular block first degree; Atrioventricular block second degree; Atrioventricular conduction time shortened; Atrioventricular dissociation; Atrioventricular node dysfunction; Bifascicular block; Bradyarrhythmia; BRASH syndrome; Brugada syndrome; Bundle branch block; Bundle branch block bilateral; Bundle branch block left; Bundle branch block right; Conduction disorder; Defect conduction intraventricular; Ectopic atrial rhythm; Electrocardiogram delta waves abnormal; Electrocardiogram PR prolongation; Electrocardiogram PR shortened; Electrocardiogram QRS complex prolonged; Electrocardiogram QT prolonged; Electrocardiogram repolarisation abnormality; Fascicular block; Ictal bradycardia syndrome; Lenegre's disease; Long QT syndrome; Nodal arrhythmia; Nodal rhythm; Paroxysmal atrioventricular block; Sinoatrial block; Sinus arrest; Sinus arrhythmia; Sinus bradycardia; Sinus node dysfunction; Trifascicular block; Ventricular asystole; Ventricular dyssynchrony; Wandering pacemaker; Wolff-Parkinson-White syndrome |
| Ventricular extrasystole | Ventricular extrasystoles; Ventricular parasystole |
| Sudden death | Sudden cardiac death; Sudden death |
| Pericarditis | Autoimmune pericarditis; Immune-mediated pericarditis; Myopericarditis; Pericarditis; Pericarditis adhesive; Pericarditis constrictive; Pleuropericarditis |
| Pericardial effusion | Cardiac tamponade; Pericardial effusion |
| Endocarditis | Abiotrophia defectiva endocarditis; Endocarditis; Endocarditis candida; Endocarditis enterococcal; Endocarditis haemophilus; Endocarditis histoplasma; Endocarditis pseudomonal; Endocarditis Q fever; Endocarditis staphylococcal; Endocarditis viral; Fungal endocarditis; Lupus endocarditis; Prosthetic valve endocarditis; Septic endocarditis; Streptococcal endocarditis; Subacute endocarditis |
| Valvular disease | Aortic annulus rupture; Aortic valve atresia; Aortic valve calcification; Aortic valve disease; Aortic valve disease mixed; Aortic valve incompetence; Aortic valve prolapse; Aortic valve sclerosis; Aortic valve stenosis; Aortic valve thickening; Bicuspid aortic valve; Bicuspid pulmonary valve; Carcinoid heart disease; Cardiac valve abscess; Cardiac valve discolouration; Cardiac valve disease; Cardiac valve fatty infiltration; Cardiac valve replacement complication; Cardiac valve rupture; Cardiac valve sclerosis; Cardiac valve thickening; Cardiac valve vegetation; Congenital aortic valve incompetence; Congenital aortic valve stenosis; Congenital heart valve disorder; Congenital heart valve incompetence; Congenital mitral valve incompetence; Congenital mitral valve stenosis; Congenital pulmonary valve atresia; Congenital pulmonary valve disorder; Congenital tricuspid valve atresia; Congenital tricuspid valve incompetence; Congenital tricuspid valve stenosis; Degenerative aortic valve disease; Degenerative mitral valve disease; Degenerative multivalvular disease; Degenerative tricuspid valve disease; Heart valve calcification; Heart valve incompetence; Heart valve stenosis; Heyde's syndrome; Ischaemic mitral regurgitation; Lambl's excrescences; Mitral face; Mitral perforation; Mitral valve atresia; Mitral valve calcification; Mitral valve disease; Mitral valve disease mixed; Mitral valve dysplasia; Mitral valve incompetence; Mitral valve prolapse; Mitral valve sclerosis; Mitral valve stenosis; Mitral valve thickening; Myxomatous mitral valve degeneration; Parachute mitral valve; Prosthetic cardiac valve regurgitation; Pulmonary valve calcification; Pulmonary valve disease; Pulmonary valve incompetence; Pulmonary valve sclerosis; Pulmonary valve stenosis; Pulmonary valve stenosis congenital; Pulmonary valve thickening; Shone complex; Straddling tricuspid valve; Subvalvular aortic stenosis; Supravalvular aortic stenosis; Systolic anterior motion of mitral valve; Tricuspid valve calcification; Tricuspid valve disease; Tricuspid valve disease mixed; Tricuspid valve incompetence; Tricuspid valve prolapse; Tricuspid valve sclerosis; Tricuspid valve stenosis; Tricuspid valve thickening; Tricuspid valve thrombosis; Unicuspid aortic valve; Williams syndrome |
| Thromboembolic disease | Acute aortic syndrome; Acute coronary syndrome; Acute myocardial infarction; Administration site thrombosis; Adrenal thrombosis; Amaurosis; Amaurosis fugax; Aneurysm thrombosis; Angiogram abnormal; Angiogram cerebral abnormal; Angiogram peripheral abnormal; Angioplasty; Angiostomy; Antiphospholipid syndrome; Aortic aneurysm thrombosis; Aortic bypass; Aortic embolus; Aortic surgery; Aortic thrombosis; Aortogram abnormal; Application site thrombosis; Arterectomy; Arterectomy with graft replacement; Arterial angioplasty; Arterial bypass operation; Arterial graft; Arterial occlusive disease; Arterial recanalisation procedure; Arterial revascularisation; Arterial stent insertion; Arterial therapeutic procedure; Arterial thrombosis; Arteriogram abnormal; Arteriogram carotid abnormal; Arteriotomy; Arteriovenous fistula occlusion; Arteriovenous fistula thrombosis; Arteriovenous graft thrombosis; Artificial blood vessel occlusion; Aseptic cavernous sinus thrombosis; Atherectomy; Atherosclerotic plaque rupture; Atrial appendage closure; Atrial appendage resection; Atrial thrombosis; Autoimmune heparin-induced thrombocytopenia; Axillary vein thrombosis; Basal ganglia infarction; Basal ganglia stroke; Basilar artery occlusion; Basilar artery thrombosis; Blindness transient; Bone infarction; Brachiocephalic artery occlusion; Brachiocephalic vein occlusion; Brachiocephalic vein thrombosis; Brain stem embolism; Brain stem infarction; Brain stem stroke; Brain stem thrombosis; Budd-Chiari syndrome; Capsular warning syndrome; Cardiac ventricular thrombosis; Carotid angioplasty; Carotid arterial embolus; Carotid artery bypass; Carotid artery occlusion; Carotid artery stent insertion; Carotid artery thrombosis; Carotid endarterectomy; Carotid revascularisation; Catheter directed thrombolysis; Catheter site thrombosis; Catheterisation venous; Cavernous sinus thrombosis; Central venous catheterisation; Cerebellar artery occlusion; Cerebellar artery thrombosis; Cerebellar embolism; Cerebellar infarction; Cerebral angioplasty; Cerebral artery embolism; Cerebral artery occlusion; Cerebral artery stent insertion; Cerebral artery thrombosis; Cerebral congestion; Cerebral hypoperfusion; Cerebral infarction; Cerebral infarction foetal; Cerebral ischaemia; Cerebral microembolism; Cerebral microinfarction; Cerebral revascularisation; Cerebral septic infarct; Cerebral thrombosis; Cerebral vascular occlusion; Cerebral venous sinus thrombosis; Cerebral venous thrombosis; Cerebrospinal thrombotic tamponade; Cerebrovascular accident; Cerebrovascular accident prophylaxis; Cerebrovascular disorder; Cerebrovascular insufficiency; Cerebrovascular operation; Cerebrovascular stenosis; Choroidal infarction; Coeliac artery occlusion; Collateral circulation; Compression garment application; Coronary angioplasty; Coronary arterial stent insertion; Coronary artery bypass; Coronary artery embolism; Coronary artery occlusion; Coronary artery reocclusion; Coronary artery surgery; Coronary artery thrombosis; Coronary bypass thrombosis; Coronary endarterectomy; Coronary revascularisation; Coronary vascular graft occlusion; Deep vein thrombosis; Deep vein thrombosis postoperative; Device embolisation; Device occlusion; Device related thrombosis; Diplegia; Directional Doppler flow tests abnormal; Disseminated intravascular coagulation; Disseminated intravascular coagulation in newborn; Embolia cutis medicamentosa; Embolic cerebellar infarction; Embolic cerebral infarction; Embolic pneumonia; Embolic stroke; Embolism; Embolism arterial; Embolism venous; Endarterectomy; Eye infarction; Femoral artery embolism; Fluorescence angiogram abnormal; Foetal cerebrovascular disorder; Foetal vascular malperfusion; Gastric infarction; Graft thrombosis; Haemorrhagic adrenal infarction; Haemorrhagic cerebral infarction; Haemorrhagic infarction; Haemorrhagic stroke; Haemorrhagic transformation stroke; Haemorrhoids thrombosed; Hemiparesis; Hemiplegia; Heparin-induced thrombocytopenia; Hepatic artery embolism; Hepatic artery occlusion; Hepatic artery thrombosis; Hepatic infarction; Hepatic vascular thrombosis; Hepatic vein embolism; Hepatic vein occlusion; Hepatic vein thrombosis; Homans' sign positive; Hypothenar hammer syndrome; Iliac artery embolism; Iliac artery occlusion; Iliac vein occlusion; Implant site thrombosis; Incision site vessel occlusion; Incomplete atrial appendage closure; Infarction; Inferior vena cava syndrome; Inferior vena caval occlusion; Infusion site thrombosis; Injection site thrombosis; Inner ear infarction; Instillation site thrombosis; Internal capsule infarction; Intestinal infarction; Intra-aortic balloon placement; Intracardiac mass; Intracardiac thrombus; Intraoperative cerebral artery occlusion; Ischaemic cerebral infarction; Ischaemic stroke; Jugular vein embolism; Jugular vein occlusion; Jugular vein thrombosis; Lacunar infarction; Lambl's excrescences; Left atrial appendage closure implant; Leriche syndrome; Mahler sign; May-Thurner syndrome; Medical device site thrombosis; Mesenteric arterial occlusion; Mesenteric arteriosclerosis; Mesenteric artery embolism; Mesenteric artery stenosis; Mesenteric artery stent insertion; Mesenteric artery thrombosis; Mesenteric vascular insufficiency; Mesenteric vascular occlusion; Mesenteric vein embolism; Mesenteric vein thrombosis; Mesenteric venous occlusion; Metabolic stroke; Microembolism; Monoparesis; Monoplegia; Muscle infarction; Myocardial infarction; Myocardial necrosis; Obstetrical pulmonary embolism; Obstructive shock; Ophthalmic artery occlusion; Ophthalmic artery thrombosis; Ophthalmic vascular thrombosis; Ophthalmic vein thrombosis; Optic nerve infarction; Ovarian vein thrombosis; Paget-Schroetter syndrome; Pancreatic infarction; Papillary muscle infarction; Paradoxical embolism; Paraneoplastic thrombosis; Paraparesis; Paraplegia; Paresis; Pelvic venous thrombosis; Penile artery occlusion; Penile vein thrombosis; Percutaneous coronary intervention; Peripheral arterial occlusive disease; Peripheral arterial reocclusion; Peripheral artery angioplasty; Peripheral artery bypass; Peripheral artery occlusion; Peripheral artery stent insertion; Peripheral artery surgery; Peripheral artery thrombosis; Peripheral embolism; Peripheral endarterectomy; Peripheral revascularisation; Peripheral vein occlusion; Peripheral vein thrombosis; Peripheral vein thrombus extension; Phlebectomy; Pituitary infarction; Placental infarction; Pneumatic compression therapy; Popliteal artery entrapment syndrome; Portal shunt; Portal shunt procedure; Portal vein cavernous transformation; Portal vein embolism; Portal vein occlusion; Portal vein thrombosis; Portosplenomesenteric venous thrombosis; Post procedural myocardial infarction; Post procedural pulmonary embolism; Post procedural stroke; Post thrombotic syndrome; Postinfarction angina; Postoperative thrombosis; Postpartum thrombosis; Postpartum venous thrombosis; Precerebral artery embolism; Precerebral artery occlusion; Precerebral artery thrombosis; Profundaplasty; Prosthetic cardiac valve thrombosis; Prosthetic vessel implantation; Pseudo-occlusion of internal carotid artery; Pulmonary angioplasty; Pulmonary artery occlusion; Pulmonary artery stent insertion; Pulmonary artery therapeutic procedure; Pulmonary artery thrombosis; Pulmonary embolism; Pulmonary endarterectomy; Pulmonary infarction; Pulmonary microemboli; Pulmonary oil microembolism; Pulmonary thrombosis; Pulmonary tumour thrombotic microangiopathy; Pulmonary vein occlusion; Pulmonary veno-occlusive disease; Pulmonary venous thrombosis; Quadriparesis; Quadriplegia; Renal-limited thrombotic microangiopathy; Renal artery angioplasty; Renal artery occlusion; Renal artery revascularisation; Renal artery thrombosis; Renal embolism; Renal infarct; Renal vascular thrombosis; Renal vein embolism; Renal vein occlusion; Renal vein thrombosis; Retinal artery embolism; Retinal artery occlusion; Retinal artery thrombosis; Retinal infarction; Retinal vascular thrombosis; Retinal vein occlusion; Retinal vein thrombosis; Revascularisation procedure; Segmental arterial mediolysis; Septic pulmonary embolism; Shunt occlusion; Shunt thrombosis; SI QIII TIII pattern; Sigmoid sinus thrombosis; Silent myocardial infarction; Spermatic vein thrombosis; Spinal artery embolism; Spinal artery thrombosis; Spinal cord infarction; Spinal stroke; Splenic artery thrombosis; Splenic embolism; Splenic infarction; Splenic thrombosis; Splenic vein occlusion; Splenic vein thrombosis; Spontaneous heparin-induced thrombocytopenia syndrome; Stoma site thrombosis; Stress cardiomyopathy; Stroke in evolution; Strokectomy; Subclavian artery embolism; Subclavian artery occlusion; Subclavian artery thrombosis; Subclavian vein occlusion; Subclavian vein thrombosis; Superficial vein thrombosis; Superior mesenteric artery syndrome; Superior sagittal sinus thrombosis; Superior vena cava occlusion; Superior vena cava syndrome; Surgical vascular shunt; Testicular infarction; Thalamic infarction; Thalamic stroke; Thrombectomy; Thromboangiitis obliterans; Thromboembolectomy; Thrombolysis; Thrombophlebitis; Thrombophlebitis migrans; Thrombophlebitis neonatal; Thrombosed varicose vein; Thrombosis; Thrombosis corpora cavernosa; Thrombosis in device; Thrombosis mesenteric vessel; Thrombosis prophylaxis; Thrombosis with thrombocytopenia syndrome; Thrombotic cerebral infarction; Thrombotic microangiopathy; Thrombotic stroke; Thrombotic thrombocytopenic purpura; Thyroid infarction; Transient ischaemic attack; Transverse sinus thrombosis; Tricuspid valve thrombosis; Truncus coeliacus thrombosis; Tumour embolism; Tumour thrombectomy; Tumour thrombosis; Ultrasonic angiogram abnormal; Ultrasound Doppler abnormal; Umbilical cord occlusion; Umbilical cord thrombosis; Vaccination site thrombosis; Vascular access site thrombosis; Vascular device occlusion; Vascular graft; Vascular graft occlusion; Vascular graft thrombosis; Vascular operation; Vascular pseudoaneurysm thrombosis; Vascular stent insertion; Vascular stent occlusion; Vascular stent thrombosis; Vasodilation procedure; Vena cava embolism; Vena cava filter insertion; Vena cava filter removal; Vena cava thrombosis; Venogram abnormal; Venoocclusive disease; Venoocclusive liver disease; Venous angioplasty; Venous occlusion; Venous operation; Venous recanalisation; Venous repair; Venous stent insertion; Venous thrombosis; Venous thrombosis in pregnancy; Venous thrombosis limb; Venous thrombosis neonatal; Vertebral artery occlusion; Vertebral artery thrombosis; Vessel puncture site occlusion; Vessel puncture site thrombosis; Visceral venous thrombosis; Visual acuity reduced transiently; Visual midline shift syndrome |
| Arterial thromboembolism | Acute aortic syndrome; Acute coronary syndrome; Acute myocardial infarction; Amaurosis; Amaurosis fugax; Aneurysm thrombosis; Angioplasty; Angiostomy; Aortic aneurysm thrombosis; Aortic bypass; Aortic embolus; Aortic surgery; Aortic thrombosis; Aortogram abnormal; Arterectomy; Arterectomy with graft replacement; Arterial angioplasty; Arterial bypass operation; Arterial graft; Arterial occlusive disease; Arterial recanalisation procedure; Arterial revascularisation; Arterial stent insertion; Arterial therapeutic procedure; Arterial thrombosis; Arteriogram abnormal; Arteriogram carotid abnormal; Arteriotomy; Atherectomy; Atherosclerotic plaque rupture; Atrial appendage closure; Atrial appendage resection; Basal ganglia infarction; Basilar artery occlusion; Basilar artery thrombosis; Blindness transient; Brachiocephalic artery occlusion; Capsular warning syndrome; Carotid angioplasty; Carotid arterial embolus; Carotid artery bypass; Carotid artery occlusion; Carotid artery stent insertion; Carotid artery thrombosis; Carotid endarterectomy; Carotid revascularisation; Cerebellar artery occlusion; Cerebellar artery thrombosis; Cerebellar embolism; Cerebral angioplasty; Cerebral artery embolism; Cerebral artery occlusion; Cerebral artery stent insertion; Cerebral artery thrombosis; Cerebral hypoperfusion; Cerebral revascularisation; Cerebral vascular occlusion; Cerebrovascular insufficiency; Cerebrovascular stenosis; Coeliac artery occlusion; Coronary angioplasty; Coronary arterial stent insertion; Coronary artery bypass; Coronary artery embolism; Coronary artery occlusion; Coronary artery reocclusion; Coronary artery surgery; Coronary artery thrombosis; Coronary endarterectomy; Coronary revascularisation; Coronary vascular graft occlusion; Embolia cutis medicamentosa; Embolism; Embolism arterial; Endarterectomy; Femoral artery embolism; Hepatic artery embolism; Hepatic artery occlusion; Hepatic artery thrombosis; Hypothenar hammer syndrome; Iliac artery embolism; Iliac artery occlusion; Incomplete atrial appendage closure; Internal capsule infarction; Intra-aortic balloon placement; Intraoperative cerebral artery occlusion; Ischaemic cerebral infarction; Ischaemic stroke; Lacunar infarction; Left atrial appendage closure implant; Leriche syndrome; Mesenteric arterial occlusion; Mesenteric arteriosclerosis; Mesenteric artery embolism; Mesenteric artery stenosis; Mesenteric artery stent insertion; Mesenteric artery thrombosis; Metabolic stroke; Myocardial infarction; Myocardial necrosis; Ophthalmic artery occlusion; Ophthalmic artery thrombosis; Papillary muscle infarction; Penile artery occlusion; Percutaneous coronary intervention; Peripheral arterial occlusive disease; Peripheral arterial reocclusion; Peripheral artery angioplasty; Peripheral artery bypass; Peripheral artery occlusion; Peripheral artery stent insertion; Peripheral artery surgery; Peripheral artery thrombosis; Peripheral embolism; Peripheral endarterectomy; Popliteal artery entrapment syndrome; Post procedural myocardial infarction; Postinfarction angina; Precerebral artery embolism; Precerebral artery occlusion; Precerebral artery thrombosis; Profundaplasty; Pseudo-occlusion of internal carotid artery; Pulmonary angioplasty; Pulmonary artery occlusion; Pulmonary artery stent insertion; Pulmonary artery therapeutic procedure; Pulmonary artery thrombosis; Pulmonary endarterectomy; Pulmonary tumour thrombotic microangiopathy; Renal-limited thrombotic microangiopathy; Renal artery angioplasty; Renal artery occlusion; Renal artery revascularisation; Renal artery thrombosis; Renal embolism; Retinal artery embolism; Retinal artery occlusion; Retinal artery thrombosis; Segmental arterial mediolysis; Silent myocardial infarction; Spinal artery embolism; Spinal artery thrombosis; Splenic artery thrombosis; Splenic embolism; Stress cardiomyopathy; Stroke in evolution; Subclavian artery embolism; Subclavian artery occlusion; Subclavian artery thrombosis; Superior mesenteric artery syndrome; Thromboembolectomy; Thrombotic microangiopathy; Thrombotic thrombocytopenic purpura; Transient ischaemic attack; Truncus coeliacus thrombosis; Vascular pseudoaneurysm thrombosis; Vertebral artery occlusion; Vertebral artery thrombosis; Visual acuity reduced transiently |
| Venous thromboembolism | Aseptic cavernous sinus thrombosis; Axillary vein thrombosis; Brachiocephalic vein occlusion; Brachiocephalic vein thrombosis; Budd-Chiari syndrome; Catheterisation venous; Cavernous sinus thrombosis; Central venous catheterisation; Cerebral venous sinus thrombosis; Cerebral venous thrombosis; Compression garment application; Deep vein thrombosis; Deep vein thrombosis postoperative; Embolism venous; Hepatic vein embolism; Hepatic vein occlusion; Hepatic vein thrombosis; Homans' sign positive; Iliac vein occlusion; Inferior vena cava syndrome; Inferior vena caval occlusion; Jugular vein embolism; Jugular vein occlusion; Jugular vein thrombosis; Mahler sign; May-Thurner syndrome; Mesenteric vein embolism; Mesenteric vein thrombosis; Mesenteric venous occlusion; Obstetrical pulmonary embolism; Obstructive shock; Ophthalmic vein thrombosis; Ovarian vein thrombosis; Paget-Schroetter syndrome; Pelvic venous thrombosis; Penile vein thrombosis; Peripheral vein occlusion; Peripheral vein thrombosis; Peripheral vein thrombus extension; Phlebectomy; Portal vein cavernous transformation; Portal vein embolism; Portal vein occlusion; Portal vein thrombosis; Portosplenomesenteric venous thrombosis; Post procedural pulmonary embolism; Post thrombotic syndrome; Postoperative thrombosis; Postpartum venous thrombosis; Pulmonary embolism; Pulmonary infarction; Pulmonary microemboli; Pulmonary oil microembolism; Pulmonary thrombosis; Pulmonary vein occlusion; Pulmonary veno-occlusive disease; Pulmonary venous thrombosis; Renal vein embolism; Renal vein occlusion; Renal vein thrombosis; Retinal vein occlusion; Retinal vein thrombosis; Septic pulmonary embolism; SI QIII TIII pattern; Sigmoid sinus thrombosis; Spermatic vein thrombosis; Splenic vein occlusion; Splenic vein thrombosis; Subclavian vein occlusion; Subclavian vein thrombosis; Superficial vein thrombosis; Superior sagittal sinus thrombosis; Superior vena cava occlusion; Superior vena cava syndrome; Thrombophlebitis; Thrombophlebitis migrans; Thrombophlebitis neonatal; Thrombosed varicose vein; Thrombosis corpora cavernosa; Transverse sinus thrombosis; Vascular graft; Vena cava embolism; Vena cava filter insertion; Vena cava filter removal; Vena cava thrombosis; Venogram abnormal; Venoocclusive disease; Venoocclusive liver disease; Venous angioplasty; Venous occlusion; Venous operation; Venous recanalisation; Venous repair; Venous stent insertion; Venous thrombosis; Venous thrombosis in pregnancy; Venous thrombosis limb; Venous thrombosis neonatal; Visceral venous thrombosis |
| Cerebrovascular disease | Central nervous system vasculitis; Cerebral arteritis; Cerebral capillary telangiectasia; Cerebral circulatory failure; Cerebral congestion; Cerebral hypoperfusion; Cerebral venous sinus thrombosis; Chronic cerebrospinal venous insufficiency; Dural arteriovenous fistula; Sigmoid sinus thrombosis; Spinal cord hypoxia; Superior sagittal sinus thrombosis; Transverse sinus thrombosis |
| Bleeding | Abdominal wall haematoma; Abdominal wall haemorrhage; Abnormal menstrual clots; Abnormal uterine bleeding; Abnormal withdrawal bleeding; Achenbach syndrome; Acute haemorrhagic leukoencephalitis; Acute haemorrhagic ulcerative colitis; Administration site bruise; Administration site haematoma; Administration site haemorrhage; Adrenal haematoma; Adrenal haemorrhage; Anal fissure haemorrhage; Anal haemorrhage; Anal ulcer haemorrhage; Anastomotic haemorrhage; Anastomotic ulcer haemorrhage; Aneurysm ruptured; Angina bullosa haemorrhagica; Anorectal varices haemorrhage; Anticoagulant-related nephropathy; Antiplatelet reversal therapy; Aortic aneurysm rupture; Aortic dissection rupture; Aortic intramural haematoma; Aortic perforation; Aortic rupture; Aponeurosis contusion; Application site bruise; Application site haematoma; Application site haemorrhage; Application site purpura; Arterial haemorrhage; Arterial intramural haematoma; Arterial perforation; Arterial rupture; Arteriovenous fistula site haematoma; Arteriovenous fistula site haemorrhage; Arteriovenous graft site haematoma; Arteriovenous graft site haemorrhage; Astringent therapy; Atrial rupture; Auricular haematoma; Basal ganglia haematoma; Basal ganglia haemorrhage; Basilar artery perforation; Bladder tamponade; Bleeding varicose vein; Blood blister; Blood loss anaemia; Blood urine; Blood urine present; Bloody discharge; Bloody peritoneal effluent; Bone contusion; Bone marrow haemorrhage; Brain contusion; Brain stem haematoma; Brain stem haemorrhage; Brain stem microhaemorrhage; Breast haematoma; Breast haemorrhage; Broad ligament haematoma; Bronchial haemorrhage; Bronchial varices haemorrhage; Bullous haemorrhagic dermatosis; Bursal haematoma; Cardiac contusion; Carotid aneurysm rupture; Carotid artery perforation; Carotid blowout syndrome; Catheter site bruise; Catheter site haematoma; Catheter site haemorrhage; Central nervous system haemorrhage; Cephalhaematoma; Cerebellar haematoma; Cerebellar haemorrhage; Cerebellar microhaemorrhage; Cerebral aneurysm perforation; Cerebral aneurysm ruptured syphilitic; Cerebral arteriovenous malformation haemorrhagic; Cerebral artery perforation; Cerebral cyst haemorrhage; Cerebral haematoma; Cerebral haemorrhage; Cerebral haemorrhage foetal; Cerebral haemorrhage neonatal; Cerebral microhaemorrhage; Cervix haematoma uterine; Cervix haemorrhage uterine; Chest wall haematoma; Choroidal haematoma; Choroidal haemorrhage; Chronic gastrointestinal bleeding; Chronic pigmented purpura; Ciliary body haemorrhage; Coital bleeding; Colonic haematoma; Conjunctival haemorrhage; Contusion; Corneal bleeding; Cullen's sign; Cystitis haemorrhagic; Deep dissecting haematoma; Diarrhoea haemorrhagic; Disseminated intravascular coagulation; Diverticulitis intestinal haemorrhagic; Diverticulum intestinal haemorrhagic; Duodenal ulcer haemorrhage; Duodenitis haemorrhagic; Ear haemorrhage; Ecchymosis; Encephalitis haemorrhagic; Enterocolitis haemorrhagic; Epidural haemorrhage; Epistaxis; Exsanguination; Extra-axial haemorrhage; Extradural haematoma; Extradural haematoma evacuation; Extravasation blood; Eye contusion; Eye haematoma; Eye haemorrhage; Eyelid bleeding; Eyelid contusion; Eyelid haematoma; Femoral artery perforation; Femoral vein perforation; Foetal-maternal haemorrhage; Fothergill sign positive; Gallbladder haematoma; Gastric haemorrhage; Gastric occult blood positive; Gastric ulcer haemorrhage; Gastric ulcer haemorrhage, obstructive; Gastric ulcer perforation; Gastric varices haemorrhage; Gastritis alcoholic haemorrhagic; Gastritis haemorrhagic; Gastroduodenal haemorrhage; Gastrointestinal anastomotic haemorrhage; Gastrointestinal haemorrhage; Gastrointestinal polyp haemorrhage; Gastrointestinal ulcer haemorrhage; Gastrointestinal vascular malformation haemorrhagic; Genital contusion; Genital haemorrhage; Gingival bleeding; Graft haemorrhage; Grey Turner's sign; Haemangioma rupture; Haemarthrosis; Haematemesis; Haematochezia; Haematocoele; Haematoma; Haematoma evacuation; Haematoma infection; Haematoma muscle; Haematosalpinx; Haematospermia; Haematotympanum; Haematuria; Haematuria traumatic; Haemobilia; Haemoperitoneum; Haemophilic arthropathy; Haemophilic pseudotumour; Haemoptysis; Haemorrhage; Haemorrhage coronary artery; Haemorrhage foetal; Haemorrhage in pregnancy; Haemorrhage intracranial; Haemorrhage neonatal; Haemorrhage subcutaneous; Haemorrhage subepidermal; Haemorrhage urinary tract; Haemorrhagic adrenal infarction; Haemorrhagic arteriovenous malformation; Haemorrhagic ascites; Haemorrhagic breast cyst; Haemorrhagic cerebellar infarction; Haemorrhagic cerebral infarction; Haemorrhagic cyst; Haemorrhagic diathesis; Haemorrhagic disease of newborn; Haemorrhagic disorder; Haemorrhagic erosive gastritis; Haemorrhagic gastroenteritis; Haemorrhagic hepatic cyst; Haemorrhagic infarction; Haemorrhagic necrotic pancreatitis; Haemorrhagic occlusive retinal vasculitis; Haemorrhagic ovarian cyst; Haemorrhagic stroke; Haemorrhagic thyroid cyst; Haemorrhagic transformation stroke; Haemorrhagic tumour necrosis; Haemorrhagic urticaria; Haemorrhagic vasculitis; Haemorrhoidal haemorrhage; Haemostasis; Haemothorax; Heavy menstrual bleeding; Henoch-Schonlein purpura; Hepatic artery haemorrhage; Hepatic haemangioma rupture; Hepatic haematoma; Hepatic haemorrhage; Hereditary haemorrhagic telangiectasia; Hyperfibrinolysis; Hypergammaglobulinaemic purpura of Waldenstrom; Hyphaema; Iliac artery perforation; Iliac artery rupture; Iliac vein perforation; Immune thrombocytopenia; Implant site bruising; Implant site haematoma; Implant site haemorrhage; Incision site haematoma; Incision site haemorrhage; Increased tendency to bruise; Induced abortion haemorrhage; Inferior vena cava perforation; Infusion site bruising; Infusion site haematoma; Infusion site haemorrhage; Injection site bruising; Injection site haematoma; Injection site haemorrhage; Instillation site bruise; Instillation site haematoma; Instillation site haemorrhage; Intermenstrual bleeding; Internal haemorrhage; Intestinal haematoma; Intestinal haemorrhage; Intestinal varices haemorrhage; Intra-abdominal haematoma; Intra-abdominal haemorrhage; Intracerebral haematoma evacuation; Intracranial haematoma; Intracranial haemorrhage neonatal; Intracranial tumour haemorrhage; Intraocular haematoma; Intrapartum haemorrhage; Intratumoural haematoma; Intraventricular haemorrhage; Intraventricular haemorrhage neonatal; Iris haemorrhage; Joint microhaemorrhage; Jugular vein haemorrhage; Kidney contusion; Lacrimal haemorrhage; Large intestinal haemorrhage; Large intestinal ulcer haemorrhage; Laryngeal haematoma; Laryngeal haemorrhage; Lip haematoma; Lip haemorrhage; Liver contusion; Lower gastrointestinal haemorrhage; Lower limb artery perforation; Lymph node haemorrhage; Mallory-Weiss syndrome; Mediastinal haematoma; Mediastinal haemorrhage; Medical device site bruise; Medical device site haematoma; Medical device site haemorrhage; Melaena; Melaena neonatal; Meningorrhagia; Menometrorrhagia; Menstrual clots; Mesenteric haematoma; Mesenteric haemorrhage; Mouth haemorrhage; Mucocutaneous haemorrhage; Mucosal haemorrhage; Muscle contusion; Muscle haemorrhage; Myocardial haemorrhage; Myocardial rupture; Naevus haemorrhage; Nail bed bleeding; Nasal septum haematoma; Neonatal gastrointestinal haemorrhage; Nephritis haemorrhagic; Nipple exudate bloody; Occult blood positive; Ocular retrobulbar haemorrhage; Oesophageal haemorrhage; Oesophageal intramural haematoma; Oesophageal ulcer haemorrhage; Oesophageal varices haemorrhage; Oesophagitis haemorrhagic; Omental haemorrhage; Optic disc haemorrhage; Optic nerve sheath haemorrhage; Oral blood blister; Oral contusion; Oral mucosa haematoma; Oral purpura; Orbital haematoma; Orbital haemorrhage; Osteorrhagia; Ovarian haematoma; Ovarian haemorrhage; Palpable purpura; Pancreatic haemorrhage; Pancreatic pseudocyst haemorrhage; Pancreatitis haemorrhagic; Papillary muscle haemorrhage; Paranasal sinus haematoma; Paranasal sinus haemorrhage; Parathyroid haemorrhage; Parotid gland haemorrhage; Pelvic haematoma; Pelvic haematoma obstetric; Pelvic haemorrhage; Penile contusion; Penile haematoma; Penile haemorrhage; Peptic ulcer haemorrhage; Pericardial haemorrhage; Perineal haematoma; Periorbital haematoma; Periorbital haemorrhage; Periosteal haematoma; Peripartum haemorrhage; Peripheral artery aneurysm rupture; Peripheral exudative haemorrhagic chorioretinopathy; Peritoneal haematoma; Periventricular haemorrhage neonatal; Petechiae; Pharyngeal contusion; Pharyngeal haematoma; Pharyngeal haemorrhage; Pituitary apoplexy; Pituitary haemorrhage; Placenta praevia haemorrhage; Plasmin increased; Polymenorrhagia; Post-traumatic punctate intraepidermal haemorrhage; Post abortion haemorrhage; Post procedural contusion; Post procedural haematoma; Post procedural haematuria; Post procedural haemorrhage; Post transfusion purpura; Postmenopausal haemorrhage; Postpartum haemorrhage; Prekallikrein increased; Premature separation of placenta; Procedural haemorrhage; Proctitis haemorrhagic; Prostatic haemorrhage; Pulmonary alveolar haemorrhage; Pulmonary contusion; Pulmonary haematoma; Pulmonary haemorrhage; Pulmonary haemorrhage neonatal; Puncture site bruise; Puncture site haematoma; Puncture site haemorrhage; Purpura; Purpura fulminans; Purpura neonatal; Purpura non-thrombocytopenic; Purpura senile; Putamen haemorrhage; Radiation associated haemorrhage; Rectal haemorrhage; Rectal ulcer haemorrhage; Renal artery perforation; Renal cyst haemorrhage; Renal haematoma; Renal haemorrhage; Respiratory tract haemorrhage; Respiratory tract haemorrhage neonatal; Retinal aneurysm rupture; Retinal haemorrhage; Retinopathy haemorrhagic; Retroperitoneal haematoma; Retroperitoneal haemorrhage; Retroplacental haematoma; Ruptured cerebral aneurysm; Scalp haematoma; Scleral haematoma; Scleral haemorrhage; Scrotal haematocoele; Scrotal haematoma; Scrotal haemorrhage; Shock haemorrhagic; Skin haemorrhage; Skin neoplasm bleeding; Skin ulcer haemorrhage; Small intestinal haemorrhage; Small intestinal ulcer haemorrhage; Soft tissue haemorrhage; Spermatic cord haemorrhage; Spinal cord haematoma; Spinal cord haemorrhage; Spinal epidural haematoma; Spinal epidural haemorrhage; Spinal subarachnoid haemorrhage; Spinal subdural haematoma; Spinal subdural haemorrhage; Spleen contusion; Splenic artery perforation; Splenic haematoma; Splenic haemorrhage; Splenic varices haemorrhage; Splinter haemorrhages; Spontaneous haematoma; Spontaneous haemorrhage; Stoma site haemorrhage; Stomatitis haemorrhagic; Subarachnoid haematoma; Subarachnoid haemorrhage; Subarachnoid haemorrhage neonatal; Subcapsular hepatic haematoma; Subcapsular renal haematoma; Subcapsular splenic haematoma; Subchorionic haematoma; Subchorionic haemorrhage; Subclavian artery perforation; Subclavian vein perforation; Subcutaneous haematoma; Subdural haematoma; Subdural haematoma evacuation; Subdural haemorrhage; Subdural haemorrhage neonatal; Subendocardial haemorrhage; Subgaleal haematoma; Subgaleal haemorrhage; Subretinal haematoma; Superior vena cava perforation; Testicular haemorrhage; Thalamus haemorrhage; Third stage postpartum haemorrhage; Thoracic haemorrhage; Thrombocytopenic purpura; Thrombomodulin increased; Thrombotic thrombocytopenic purpura; Thyroid haemorrhage; Tongue haematoma; Tongue haemorrhage; Tonsillar haemorrhage; Tooth pulp haemorrhage; Tooth socket haemorrhage; Tracheal haemorrhage; Traumatic haematoma; Traumatic haemorrhage; Traumatic haemothorax; Traumatic intracranial haematoma; Traumatic intracranial haemorrhage; Tumour haemorrhage; Ulcer haemorrhage; Umbilical cord haemorrhage; Umbilical haematoma; Umbilical haemorrhage; Unexpected vaginal bleeding on hormonal IUD; Upper gastrointestinal haemorrhage; Ureteric haemorrhage; Urethral haemorrhage; Urinary bladder haematoma; Urinary bladder haemorrhage; Urinary occult blood; Urinary occult blood positive; Urogenital haemorrhage; Uterine haematoma; Uterine haemorrhage; Vaccination site bruising; Vaccination site haematoma; Vaccination site haemorrhage; Vaginal haematoma; Vaginal haemorrhage; Varicose vein ruptured; Vascular access site bruising; Vascular access site haematoma; Vascular access site haemorrhage; Vascular access site rupture; Vascular anastomotic haemorrhage; Vascular graft haemorrhage; Vascular pseudoaneurysm ruptured; Vascular purpura; Vascular rupture; Vein rupture; Venous haemorrhage; Venous perforation; Ventricle rupture; Vertebral artery perforation; Vessel puncture site bruise; Vessel puncture site haematoma; Vessel puncture site haemorrhage; Vitreous haematoma; Vitreous haemorrhage; Vocal cord haemorrhage; Vulval haematoma; Vulval haematoma evacuation; Vulval haemorrhage; Withdrawal bleed; Wound haematoma; Wound haemorrhage |
| Shock | Acute left ventricular failure; Adams-Stokes syndrome; Algid malaria; Anaphylactic reaction; Anaphylactic shock; Anaphylactic transfusion reaction; Anaphylactoid reaction; Anaphylactoid shock; Atrial parasystole; Cardiac arrest; Cardiac arrest neonatal; Cardiac death; Cardiac fibrillation; Cardiac flutter; Cardio-respiratory arrest; Cardio-respiratory arrest neonatal; Cardiogenic shock; Cardiovascular insufficiency; Circulatory collapse; CT hypotension complex; Dengue shock syndrome; Distributive shock; Electrocardiogram QT interval abnormal; Electrocardiogram QT prolonged; Endotoxic shock; Hypovolaemic shock; Long QT syndrome; Long QT syndrome congenital; Neurogenic shock; Obstructive shock; Procedural shock; Pulse absent; Pulseless electrical activity; Septic shock; Shock; Shock haemorrhagic; Shock hypoglycaemic; Shock symptom; Sudden cardiac death; Torsade de pointes; Toxic shock syndrome; Toxic shock syndrome staphylococcal; Toxic shock syndrome streptococcal; Ventricular asystole; Ventricular fibrillation; Ventricular flutter; Ventricular parasystole; Ventricular tachyarrhythmia; Ventricular tachycardia |
| Hypotension | CT hypotension complex; Dialysis hypotension; Diastolic hypotension; Hypotension; Neonatal hypotension; Orthostatic hypotension; Post procedural hypotension; Procedural hypotension |
| Hypertension | Accelerated hypertension; Blood pressure ambulatory increased; Blood pressure diastolic increased; Blood pressure inadequately controlled; Blood pressure increased; Blood pressure management; Blood pressure orthostatic increased; Blood pressure systolic increased; Catecholamine crisis; Dialysis induced hypertension; Diastolic hypertension; Eclampsia; Endocrine hypertension; Essential hypertension; Gestational hypertension; HELLP syndrome; Hyperaldosteronism; Hypertension; Hypertension neonatal; Hypertensive angiopathy; Hypertensive cardiomegaly; Hypertensive cardiomyopathy; Hypertensive cerebrovascular disease; Hypertensive crisis; Hypertensive emergency; Hypertensive encephalopathy; Hypertensive end-organ damage; Hypertensive heart disease; Hypertensive nephropathy; Hypertensive urgency; Labile hypertension; Malignant hypertension; Malignant hypertensive heart disease; Malignant renal hypertension; Maternal hypertension affecting foetus; Mean arterial pressure increased; Metabolic syndrome; Neurogenic hypertension; Orthostatic hypertension; Page kidney; Postoperative hypertension; Pre-eclampsia; Prehypertension; Primary hyperaldosteronism; Procedural hypertension; Renal artery revascularisation; Renal hypertension; Renal sympathetic nerve ablation; Renovascular hypertension; Retinopathy hypertensive; Secondary aldosteronism; Secondary hypertension; Superimposed pre-eclampsia; Supine hypertension; Syndrome Z; Systolic hypertension; White coat hypertension; Withdrawal hypertension |
| Vasculitis | Acute haemorrhagic oedema of infancy; Administration site vasculitis; Anti-neutrophil cytoplasmic antibody positive vasculitis; Aortitis; Application site vasculitis; Arteritis; Arteritis coronary; Behcet's syndrome; Capillaritis; Central nervous system vasculitis; Cerebral arteritis; Chronic pigmented purpura; Cogan's syndrome; Cutaneous vasculitis; Diabetic arteritis; Diffuse vasculitis; Eosinophilic granulomatosis with polyangiitis; Erythema induratum; Giant cell arteritis; Granulomatosis with polyangiitis; Haemorrhagic occlusive retinal vasculitis; Haemorrhagic vasculitis; Henoch-Schonlein purpura; Henoch-Schonlein purpura nephritis; Hypersensitivity vasculitis; Infusion site vasculitis; Injection site vasculitis; Kawasaki's disease; Langerhans' cell histiocytosis; Lupus vasculitis; MAGIC syndrome; Medical device site vasculitis; Microscopic polyangiitis; Nodular vasculitis; Ocular vasculitis; Polyarteritis nodosa; Polymyalgia rheumatica; Pseudovasculitis; Pulmonary vasculitis; Radiation vasculitis; Renal arteritis; Renal vasculitis; Retinal occlusive vasculitis; Retinal vasculitis; Rheumatoid vasculitis; Segmented hyalinising vasculitis; Takayasu's arteritis; Thromboangiitis obliterans; Type 2 lepra reaction; Urticarial vasculitis; Vaccination site vasculitis; Vascular purpura; Vasculitic rash; Vasculitis; Vasculitis gastrointestinal; Vasculitis necrotising; Viral vasculitis |
| Dyslipidemia | Acquired mixed hyperlipidaemia; Apolipoprotein B/Apolipoprotein A-1 ratio increased; Autoimmune hyperlipidaemia; Blood cholesterol abnormal; Blood cholesterol decreased; Blood cholesterol esterase increased; Blood cholesterol increased; Blood triglycerides abnormal; Blood triglycerides decreased; Blood triglycerides increased; Diabetic dyslipidaemia; Dyslipidaemia; Familial high density lipoprotein deficiency; Familial hypertriglyceridaemia; Fat overload syndrome; High density lipoprotein abnormal; High density lipoprotein decreased; High density lipoprotein increased; Hypercholesterolaemia; Hyperlipidaemia; Hypertriglyceridaemia; Hypertriglyceridaemic waist phenotype; Hypo HDL cholesterolaemia; Hypotriglyceridaemia; Intermediate density lipoprotein decreased; Intermediate density lipoprotein increased; LDL/HDL ratio decreased; LDL/HDL ratio increased; Lecithin-cholesterol acyltransferase deficiency; Lipid metabolism disorder; Lipids abnormal; Lipids decreased; Lipids increased; Lipoprotein (a) abnormal; Lipoprotein (a) decreased; Lipoprotein (a) increased; Lipoprotein abnormal; Lipoprotein increased; Lipoprotein metabolism disorder; Low density lipoprotein abnormal; Low density lipoprotein decreased; Low density lipoprotein increased; Metabolic syndrome; Non-high-density lipoprotein cholesterol decreased; Non-high-density lipoprotein cholesterol increased; Primary hypercholesterolaemia; Remnant-like lipoprotein particles increased; Remnant hyperlipidaemia; Total cholesterol/HDL ratio abnormal; Total cholesterol/HDL ratio decreased; Total cholesterol/HDL ratio increased; Type I hyperlipidaemia; Type II hyperlipidaemia; Type IIa hyperlipidaemia; Type IIb hyperlipidaemia; Type III hyperlipidaemia; Type IV hyperlipidaemia; Type V hyperlipidaemia; Very low density lipoprotein abnormal; Very low density lipoprotein decreased; Very low density lipoprotein increased |
| Disseminated intravascular coagulation | Disseminated intravascular coagulation |
| Cytokine release syndrome | Cytokine release syndrome; Cytokine storm |

| Table S2. Proportions and Adjusted Reporting Odds Ratios of Cardiovascular Adverse Events Associated with BsAb Therapies. | | | | | | | | | | | | | | | | | | | | |
| --- | --- | --- | --- | --- | --- | --- | --- | --- | --- | --- | --- | --- | --- | --- | --- | --- | --- | --- | --- | --- |
|  | BsAb Overall | | | | | Mosunetuzumab | | | | | Glofitamab | | | | | Epcoritamab | | | | |
| Outcome | aROR [95% CI] | N | Fatality (%)* | With CRS (%) | With CV disease (%) | aROR [95% CI] | N | Fatality (%)* | With CRS (%) | With CV disease (%) | aROR [95% CI] | N | Fatality (%)* | With CRS (%) | With CV disease (%) | aROR [95% CI] | N | Fatality (%)* | With CRS (%) | With CV disease (%) |
| CVAE | 0.77 [0.66 to 0.89] | 336 | 39.76 | 40.18 | 13.1 | 0.59 [0.43 to 0.80] | 48 | 15.22 | 25 | 14.58 | 0.74 [0.58 to 0.93] | 94 | 41.94 | 35.11 | 11.7 | 0.89 [0.75 to 1.06] | 194 | 44.56 | 46.39 | 13.4 |
| Fatal CVAE | 2.04 [1.60 to 2.60] | 132 | 100 | 42.42 | 8.33 | 0.57 [0.27 to 1.21] | 7 | 100 | 0 | 14.29 | 1.46 [1.02 to 2.08] | 39 | 100 | 23.08 | 0 | 2.26 [1.72 to 2.98] | 86 | 100 | 54.65 | 11.63 |
| Heart failure | 0.89 [0.55 to 1.43] | 24 | 66.67 | 45.83 | 12.5 | 0.80 [0.30 to 2.17] | 4 | 25 | 25 | 25 | 0.47 [0.17 to 1.29] | 4 | 75 | 25 | 0 | 1.16 [0.67 to 2.01] | 16 | 75 | 56.25 | 12.5 |
| Myocarditis | 0.45 [0.06 to 3.56] | 1 | 0 | 0 | 0 | N/A | 0 | N/A | N/A | 0 | 1.55 [0.20 to 12.20] | 1 | 0 | 0 | 0 | N/A | 0 | N/A | N/A | 0 |
| Shock | 1.14 [0.83 to 1.57] | 62 | 69.35 | 32.26 | 11.29 | 0.69 [0.31 to 1.57] | 6 | 66.67 | 16.67 | 16.67 | 1.04 [0.64 to 1.68] | 20 | 70 | 35 | 5 | 1.25 [0.85 to 1.83] | 36 | 69.44 | 33.33 | 13.89 |
| Hypotension | 2.15 [1.48 to 3.13] | 52 | 26 | 53.85 | 5.77 | 1.24 [0.55 to 2.82] | 6 | 20 | 50 | 0 | 1.26 [0.66 to 2.41] | 11 | 10 | 45.45 | 9.09 | 2.37 [1.55 to 3.62] | 35 | 31.43 | 57.14 | 5.71 |
| Coronary disease | 0.69 [0.35 to 1.36] | 11 | 27.27 | 45.45 | 18.18 | 0.87 [0.28 to 2.73] | 3 | 0 | 66.67 | 0 | 0.77 [0.24 to 2.53] | 3 | 33.33 | 0 | 33.33 | 0.62 [0.24 to 1.60] | 5 | 40 | 60 | 20 |
| Myocardial infarction | 0.82 [0.38 to 1.76] | 9 | 33.33 | 33.33 | 22.22 | 0.84 [0.21 to 3.39] | 2 | 0 | 50 | 0 | 1.12 [0.34 to 3.73] | 3 | 33.33 | 0 | 33.33 | 0.66 [0.23 to 1.90] | 4 | 50 | 50 | 25 |
| Tachyarrhythmia | 1.19 [0.78 to 1.82] | 37 | 22.22 | 40.54 | 16.22 | 2.60 [1.44 to 4.72] | 12 | 18.18 | 16.67 | 0 | 0.40 [0.15 to 1.11] | 4 | 25 | 100 | 50 | 1.13 [0.69 to 1.86] | 21 | 23.81 | 42.86 | 19.05 |
| Atrial fibrillation or flutter | 1.21 [0.71 to 2.06] | 24 | 20.83 | 41.67 | 12.5 | 3.04 [1.53 to 6.03] | 9 | 22.22 | 22.22 | 0 | 0.33 [0.08 to 1.35] | 2 | 50 | 100 | 0 | 1.00 [0.53 to 1.87] | 13 | 15.38 | 46.15 | 23.08 |
| Supraventricular Tachycardia | 1.48 [0.94 to 2.34] | 34 | 23.53 | 44.12 | 17.65 | 2.72 [1.42 to 5.21] | 10 | 20 | 20 | 0 | 0.52 [0.19 to 1.43] | 4 | 25 | 100 | 50 | 1.38 [0.82 to 2.33] | 20 | 25 | 45 | 20 |
| Ventricular tachyarrhythmia | 0.46 [0.15 to 1.42] | 4 | 0 | 25 | 0 | 0.97 [0.13 to 7.00] | 1 | 0 | 0 | 0 | N/A | 0 | N/A | N/A | 0 | 0.73 [0.21 to 2.52] | 3 | 0 | 33.33 | 0 |
| Ventricular tachycardia | 0.55 [0.14 to 2.18] | 3 | 0 | 33.33 | 0 | 1.66 [0.22 to 12.32] | 1 | 0 | 0 | 0 | N/A | 0 | N/A | N/A | 0 | 0.68 [0.15 to 3.20] | 2 | 0 | 50 | 0 |
| Ventricular fibrillation | N/A | 0 | N/A | N/A | 0 | N/A | 0 | N/A | N/A | 0 | N/A | 0 | N/A | N/A | 0 | N/A | 0 | N/A | N/A | 0 |
| Ventricular extrasystole | N/A | 0 | N/A | N/A | 0 | N/A | 0 | N/A | N/A | 0 | N/A | 0 | N/A | N/A | 0 | N/A | 0 | N/A | N/A | 0 |
| QT Prolongation | N/A | 0 | N/A | N/A | 0 | N/A | 0 | N/A | N/A | 0 | N/A | 0 | N/A | N/A | 0 | N/A | 0 | N/A | N/A | 0 |
| Bradyarrhythmia | 0.22 [0.03 to 1.66] | 1 | 0 | 0 | 0 | N/A | 0 | N/A | N/A | 0 | N/A | 0 | N/A | N/A | 0 | 0.37 [0.05 to 2.88] | 1 | 0 | 0 | 0 |
| Sudden death | N/A | 0 | N/A | N/A | 0 | N/A | 0 | N/A | N/A | 0 | N/A | 0 | N/A | N/A | 0 | N/A | 0 | N/A | N/A | 0 |
| Pericarditis | 2.01 [0.44 to 9.07] | 2 | 0 | 0 | 0 | 2.75 [0.37 to 20.18] | 1 | 0 | 0 | 0 | N/A | 0 | N/A | N/A | 0 | 1.86 [0.21 to 16.82] | 1 | 0 | 0 | 0 |
| Pericardial effusion | 0.65 [0.14 to 2.91] | 2 | 0 | 0 | 0 | N/A | 0 | N/A | N/A | 0 | 1.01 [0.13 to 7.84] | 1 | 0 | 0 | 0 | 0.73 [0.09 to 5.69] | 1 | 0 | 0 | 0 |
| Valvular disease | 0.67 [0.08 to 5.33] | 1 | 0 | 0 | 100 | N/A | 0 | N/A | N/A | 0 | N/A | 0 | N/A | N/A | 0 | 1.44 [0.17 to 12.52] | 1 | 0 | 0 | 100 |
| Endocarditis | 0.64 [0.06 to 6.56] | 1 | 0 | 100 | 0 | N/A | 0 | N/A | N/A | 0 | N/A | 0 | N/A | N/A | 0 | 1.91 [0.19 to 19.10] | 1 | 0 | 100 | 0 |
| Thromboembolic disease | 0.55 [0.40 to 0.77] | 49 | 24.49 | 32.65 | 14.29 | 0.78 [0.41 to 1.47] | 10 | 20 | 30 | 10 | 0.40 [0.20 to 0.79] | 9 | 11.11 | 33.33 | 22.22 | 0.75 [0.50 to 1.12] | 30 | 30 | 33.33 | 13.33 |
| Arterial thromboembolism | 0.75 [0.42 to 1.33] | 17 | 29.41 | 35.29 | 17.65 | 1.46 [0.65 to 3.31] | 6 | 33.33 | 50 | 16.67 | 0.50 [0.16 to 1.63] | 3 | 33.33 | 0 | 33.33 | 0.67 [0.32 to 1.44] | 8 | 25 | 37.5 | 12.5 |
| Venous thromboembolism | 0.49 [0.26 to 0.93] | 12 | 16.67 | 33.33 | 16.67 | 0.61 [0.15 to 2.46] | 2 | 0 | 0 | 0 | 0.30 [0.07 to 1.23] | 2 | 0 | 0 | 50 | 0.80 [0.38 to 1.70] | 8 | 25 | 50 | 12.5 |
| Bleeding | 0.56 [0.41 to 0.76] | 58 | 56.9 | 43.1 | 15.52 | 0.16 [0.05 to 0.50] | 3 | 0 | 0 | 66.67 | 0.69 [0.43 to 1.11] | 20 | 45 | 25 | 10 | 0.70 [0.48 to 1.01] | 35 | 68.57 | 57.14 | 14.29 |
| Cerebrovascular disease | N/A | 0 | N/A | N/A | 0 | N/A | 0 | N/A | N/A | 0 | N/A | 0 | N/A | N/A | 0 | N/A | 0 | N/A | N/A | 0 |
| Vasculitis | N/A | 0 | N/A | N/A | 0 | N/A | 0 | N/A | N/A | 0 | N/A | 0 | N/A | N/A | 0 | N/A | 0 | N/A | N/A | 0 |
| Hypertension | 0.25 [0.12 to 0.50] | 9 | 25 | 44.44 | 22.22 | N/A | 0 | N/A | N/A | 0 | 0.35 [0.11 to 1.13] | 3 | 0 | 66.67 | 0 | 0.36 [0.15 to 0.84] | 6 | 33.33 | 33.33 | 33.33 |
| Dyslipidemia | 0.17 [0.02 to 1.31] | 1 | 0 | 0 | 0 | N/A | 0 | N/A | N/A | 0 | 0.69 [0.09 to 5.25] | 1 | 0 | 0 | 0 | N/A | 0 | N/A | N/A | 0 |
| Disseminated intravascular coagulation | 0.59 [0.15 to 2.30] | 3 | 66.67 | 66.67 | 33.33 | N/A | 0 | N/A | N/A | 0 | N/A | 0 | N/A | N/A | 0 | 1.53 [0.40 to 5.85] | 3 | 66.67 | 66.67 | 33.33 |

Abbreviations: CVAE: Cardiovascular adverse events; BsAb: Bispecific Antibodies

*These proportions are based on the subset of events which had outcome data available.

## Table S3. Association Between BsAb Exposure and CVAEs Stratified by Disease.

| Outcome | DLBCL | FL | Other B-cell | B-cell unspecified | Unknown | Interaction P |
| --- | --- | --- | --- | --- | --- | --- |
| CVAE | 0.96 [0.78-1.18] | 0.99 [0.65-1.49] | 0.43 [0.15-1.26] | 0.62 [0.24-1.62] | 0.52 [0.39-0.68] | 0.003 |
| Fatal CVAE | 3.02 [2.15-4.26] | 1.3 [0.48-3.51] | 1.29 [0.28-5.87] | 1.06 [0.22-5.08] | 1.14 [0.65-1.99] | 0.016 |
| Hypotension | 2.12 [1.18-3.82] | 2.84 [1.13-7.13] | 4.04 [0.44-37.5] | 11.84 [2.04-68.72] | 1.39 [0.66-2.95] | 0.200 |
| Shock | 1.3 [0.86-1.99] | 2.52 [0.93-6.81] | NE | 1.07 [0.22-5.11] | 0.65 [0.29-1.45] | 0.111 |
| SVT | 1.4 [0.74-2.68] | 1.07 [0.32-3.51] | NE | NE | 2.13 [1.05-4.31] | 0.492 |
| AF | 0.77 [0.36-1.63] | 1.56 [0.43-5.61] | NE | NE | 2.28 [1.07-4.84] | 0.254 |
| Tachyarrhythmia | 0.96 [0.53-1.72] | 1.69 [0.63-4.52] | NE | NE | 1.63 [0.81-3.3] | 0.420 |
| Values are adjusted reporting odds ratios [95% CI] | | | | | | |
| NE = not estimable due to sparse data | | | | | | |
| Interaction P from likelihood ratio test comparing models with and without BsAb × disease category interaction | | | | | | |
| Models adjusted for age (restricted cubic splines), sex, BTK inhibitor, anthracycline, cyclophosphamide, and CAR-T exposure | | | | | | |
| DLBCL = diffuse large B-cell lymphoma; FL = follicular lymphoma | | | | | | |

## Table S4. Sensitivity Analysis Excluding Reports with Concomitant Cardiotoxic Drugs Reported.

| Outcome | N (Primary) | aROR [95% CI] (Primary) | N (Excl. Cardiotoxic) | aROR [95% CI] (Excl. Cardiotoxic) |
| --- | --- | --- | --- | --- |
| CVAE | 336 | 0.77 [0.66-0.89] | 274 | 0.74 [0.62-0.88] |
| Fatal CVAE | 132 | 2.04 [1.6-2.6] | 108 | 1.55 [1.11-2.15] |
| Hypotension | 52 | 2.15 [1.48-3.13] | 45 | 2.26 [1.44-3.55] |
| Shock | 62 | 1.14 [0.83-1.57] | 44 | 1.03 [0.67-1.59] |
| AF | 24 | 1.21 [0.71-2.06] | 22 | 2.22 [1.21-4.09] |
| SVT | 34 | 1.48 [0.94-2.34] | 28 | 2.13 [1.22-3.71] |
| Tachyarrhythmia | 37 | 1.19 [0.78-1.82] | 31 | 1.82 [1.09-3.03] |
| aROR = adjusted reporting odds ratio; CI = confidence interval | | | | |
| Primary analysis: all reports, adjusted for concomitant cardiotoxic drugs (anthracycline, cyclophosphamide, BTK inhibitor, CAR-T) | | | | |
| Sensitivity analysis: restricted to reports without concomitant cardiotoxic drug exposure (86.8% of BsAb reports, 97.7% of non-BsAb reports) | | | | |
| Both analyses adjusted for age (restricted cubic splines), sex, and disease category | | | | |

## Table S5. Sensitivity Analysis Excluding Cases with Concurrent CRS

| Outcome | N (Primary) | aROR [95% CI] (Primary) | N (Excl. CRS) | aROR [95% CI] (Excl. CRS) |
| --- | --- | --- | --- | --- |
| CVAE | 336 | 0.77 [0.66-0.89] | 201 | 0.47 [0.39-0.55] |
| Fatal CVAE | 132 | 2.04 [1.6-2.6] | 76 | 1.33 [0.99-1.79] |
| Hypotension | 52 | 2.15 [1.48-3.13] | 24 | 1.16 [0.69-1.95] |
| Shock | 62 | 1.14 [0.83-1.57] | 42 | 0.89 [0.61-1.29] |
| aROR = adjusted reporting odds ratio; CI = confidence interval; CRS = cytokine release syndrome | | | | |
| Primary analysis includes all cases; sensitivity analysis excludes cases with concurrent CRS reporting | | | | |
| Models adjusted for age (restricted cubic splines), sex, disease category, BTK inhibitor, anthracycline, cyclophosphamide, and CAR-T exposure | | | | |

##
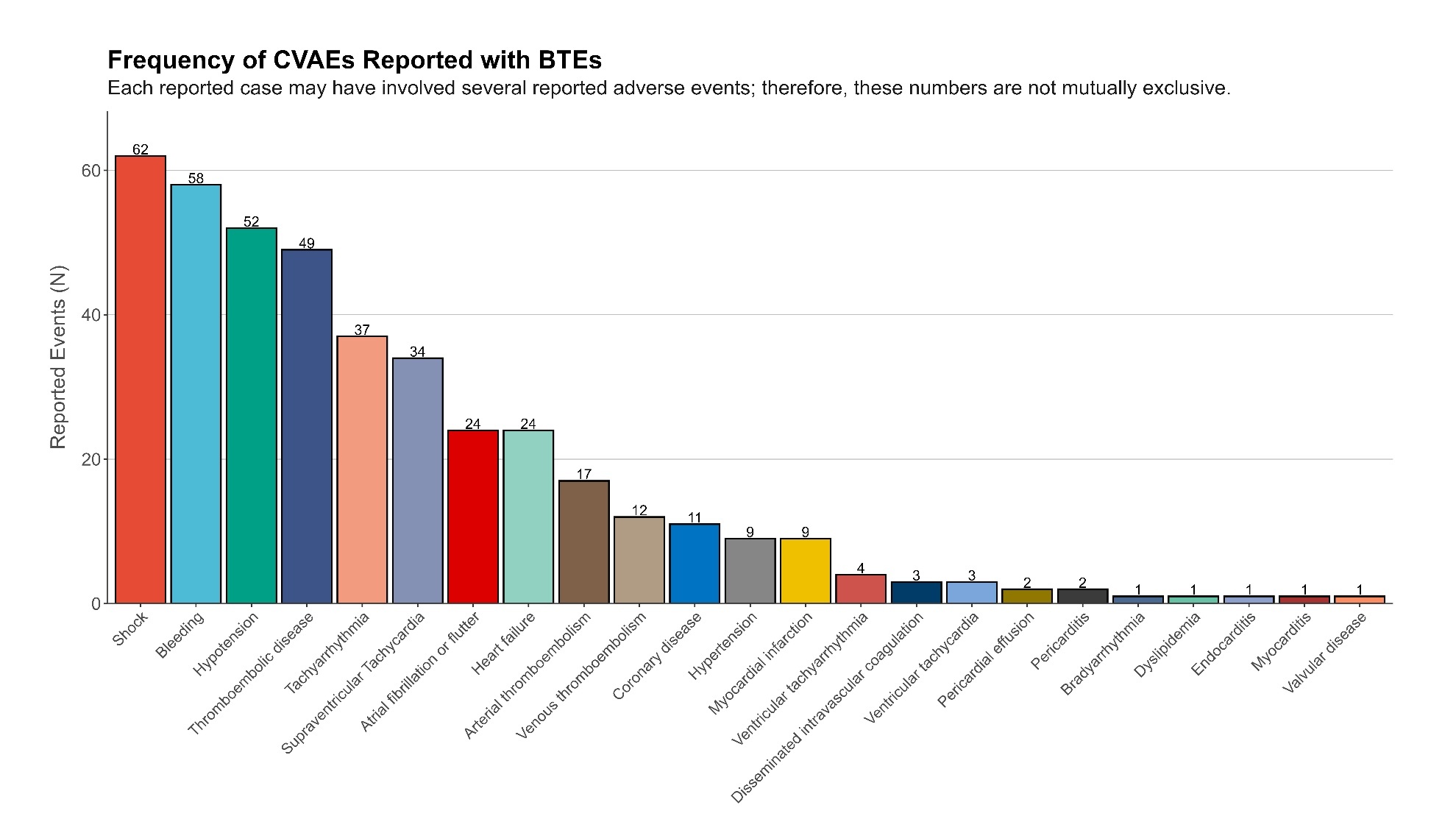
Figure S1. Frequency of Cardiovascular Adverse Events Reported with Bispecific Antibodies (2022-2025).

## Figure S2. Fatality Rate of Cardiovascular Adverse Events Reported with Bispecific Antibodies.


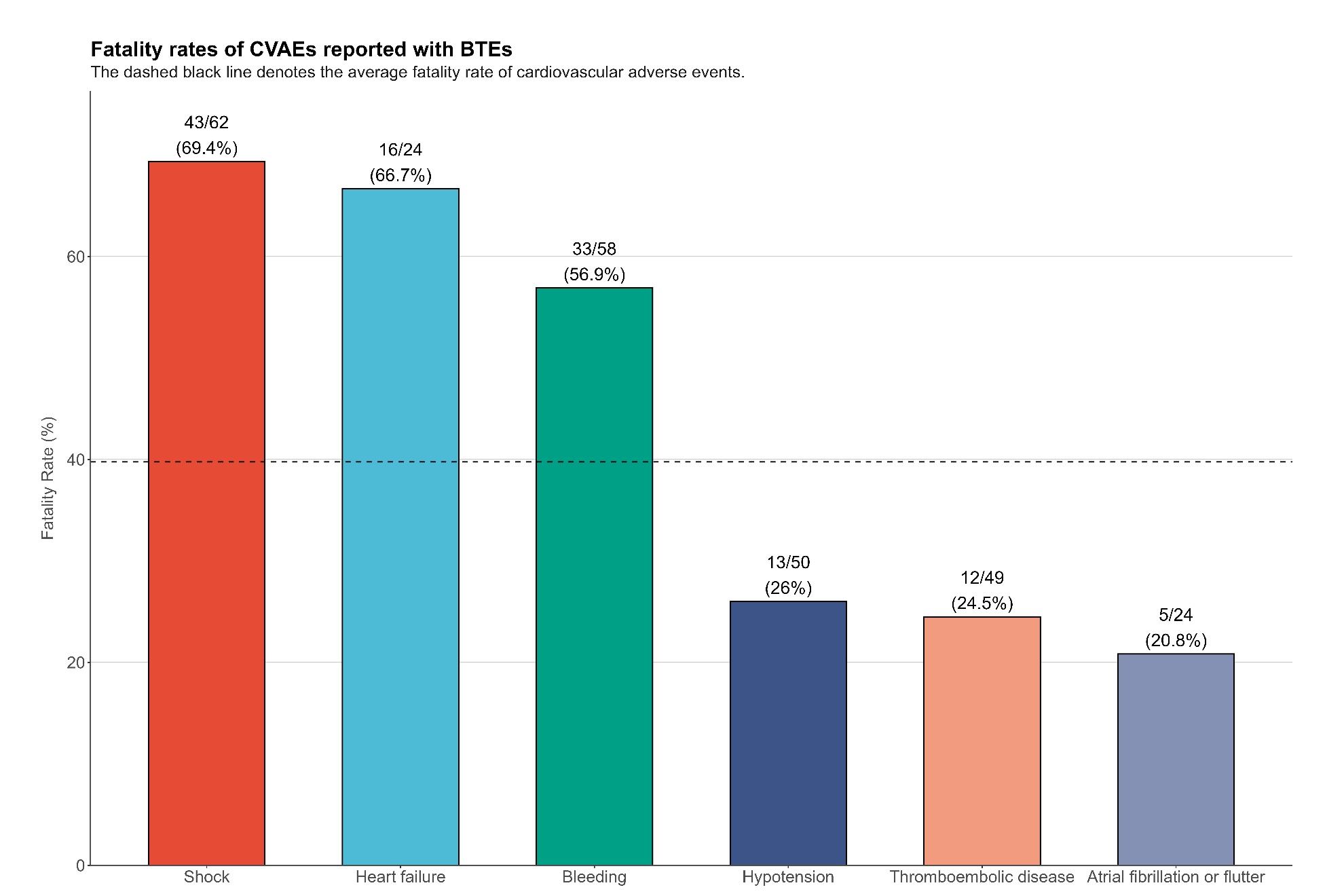


##
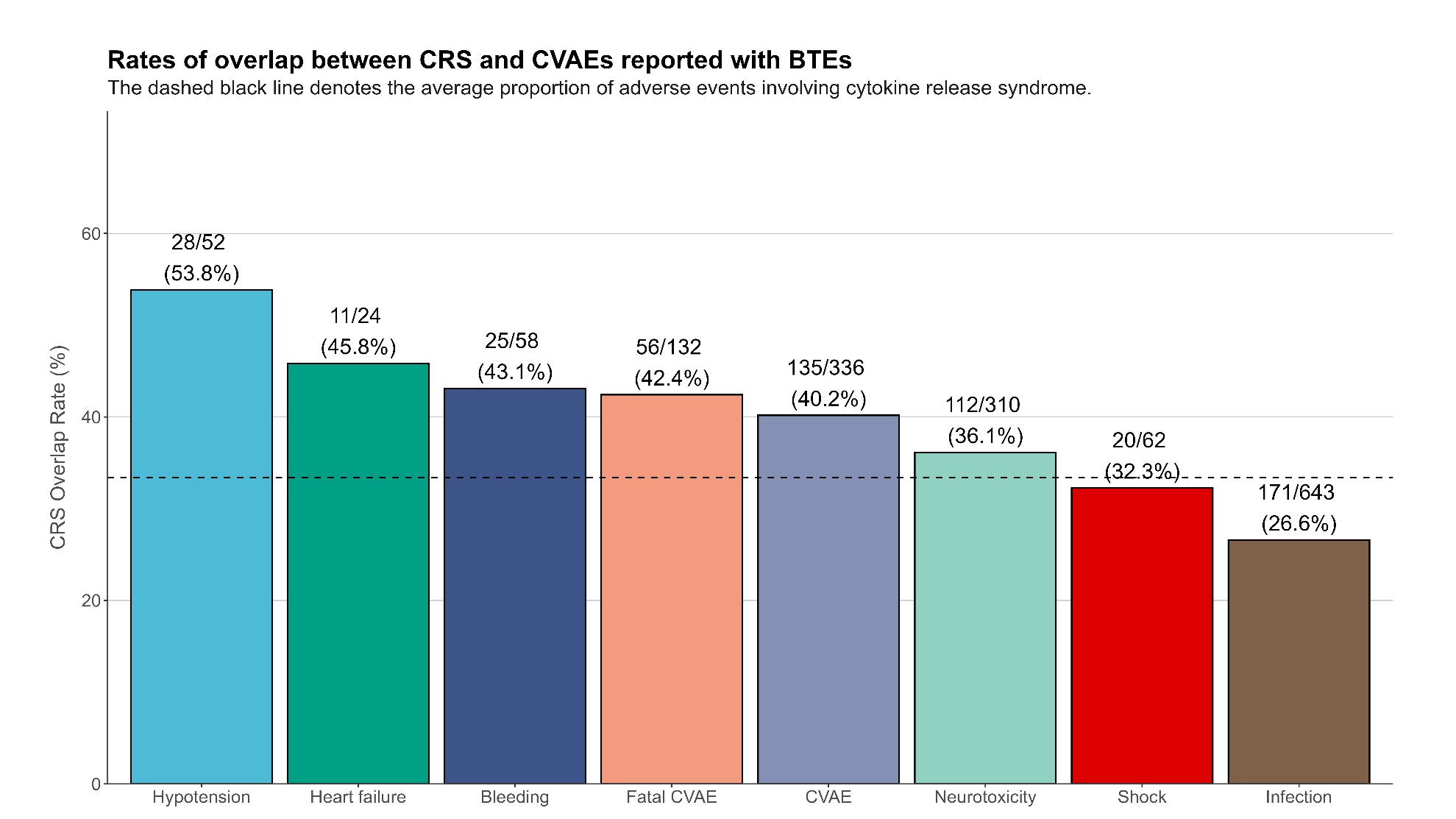
Figure S3. Overlap Between Cardiovascular Adverse Events and Cytokine Release Syndrome Reported with Bispecific Antibodies.
